# Navigated 2-level posterior lumbar fusion: a 5-cm-incision procedure

**DOI:** 10.1186/s13018-015-0338-x

**Published:** 2016-01-04

**Authors:** Yu Wang, Hong Liu, Yongkai Hu, Xiaodong Yi, Chunde Li

**Affiliations:** Department of Orthopaedics, Peking University First Hospital, Xicheng District, Beijing, 100034 China

**Keywords:** Posterior lumbar fusion, PLF, Minimally invasive, Percutaneous, Navigated surgery, Length of incision

## Abstract

**Background:**

The current study presents a technique (navigated posterior lumbar fusion) which takes a 5-cm incision to accomplish a 2-level posterior lumbar fusion (PLF) and compared its efficacy and efficiency with those of conventional PLF.

**Methods:**

Forty patients who were indicated for 2-level lumbar fusion were included and randomized to either navigated PLF group or conventional PLF group. Blood loss, operation time, incision length, complications, bed rest period, and length of hospitalization were recorded. Oswestry Disability Index (ODI) scoring was also performed for each patient before surgery, 3 months after surgery, and 2 years after surgery.

**Results:**

The incision length was significantly shorter in the navigated PLF group than in the conventional PLF group (4.8 vs. 10.9 cm, *p* = 0.001). Accordingly, the blood loss was also significantly less in the navigated PLF group than in the conventional PLF group (209.0 vs. 334.0 ml, *p* = 0.047). There was no significant difference in total operation time between the two groups (160.7 vs. 144.4 min, *p* = 0.116). Compared to the conventional PLF group, the navigated PLF group showed significantly less postoperative blood loss, less time to mobilization, and shorter length of hospital stay. The ODI score improved significantly in the both groups immediately after surgery, and maintained well in the following 2 years.

**Conclusion:**

Compared to conventional PLF, navigated PLF proved to be superior with regard to incision length, blood loss, time to mobilization, and shorter length of hospital stay.

## Background

Posterior lumbar fusion (PLF) is a commonly performed spine surgery. The length of incision for a 2-level PLF usually ranges from 8 to 12 cm, depending on not only patient’s size but also surgeon’s preference and skills. Such techniques as percutaneous pedicle screw placement and expandable retractor system have been applied in PLF procedures in the last decade, aiming to lessen approach-related morbidity. However, such minimally invasive techniques always require totally four to seven incisions, and one of these incisions has to be around 4 cm in length so that an expandable retractor can be inserted [1–9].

With the advance navigation technology, navigated posterior lumbar fusion (navigated PLF) has become a new option for spine surgeons. The current paper presents a technique which needs only a 5-cm incision to accomplish a 2-level PLF.

## Methods

This study was reviewed and approved by the Institutional Review Board of Peking University.

### Navigated PLF

The indications for navigated PLF were symptomatic 2-level degenerative disc disease. Navigated PLF takes one incision, the length of which is about 5 cm. Through the 5-cm incision, pedicle screw placement, decompression, discectomy, cage insertion, and bone grafting can be performed. Accordingly, because of the incision’s being small, blood loss can be decreased. Another advantage of navigated PLF is that the pedicle screws are inserted under the guidance of the infra-red navigators, which not only make the procedure safer but also completely avoid the operation personnel’s exposure to radiation.

### Surgical techniques of navigated PLF

The indications for conventional PLF were the same as those of navigated PLF. Navigated PLF is performed with the patient under general anesthesia and in prone position on a carbon-fiber operating table. A 5-cm longitudinal median incision is made. Detachment of paravertebral muscles and exposure of laminas are performed bilaterally.

**Fig. 1:**
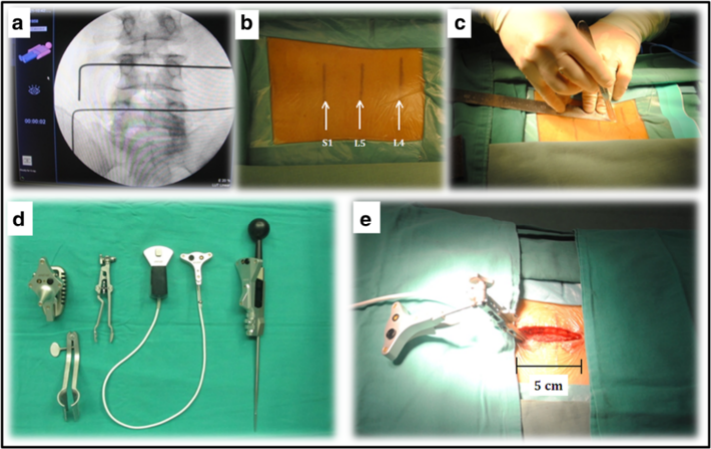
**a** The pedicles of L4, L5, and S1 are located with fluoroscopy. **b** Three transversal lines (*arrows*) are drawn on the skin to mark the location of the pedicles of L4, L5, and S1. **c** A 5-cm longitudinal median incision is made. **d** The navigated instruments are used in the procedure. **e** A patient tracker is fixed to the spinal process after exposure

**Fig. 2:**
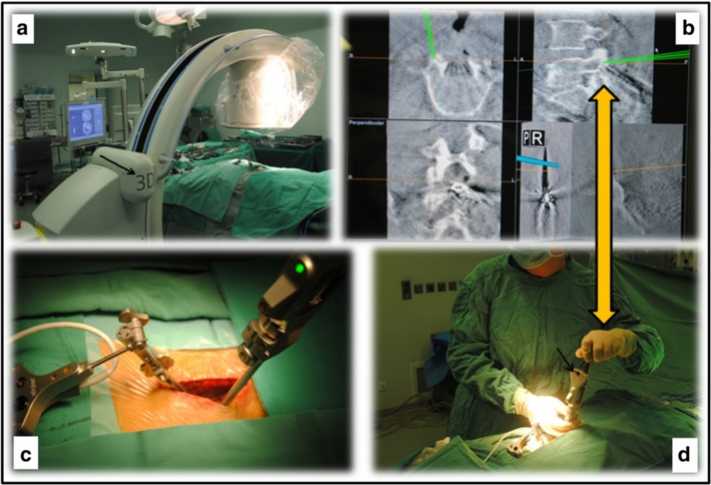
**a** Once a patient tracker has been fixed to the spinal process, a scanning will be performed with a 3D C-arm (*arrow*). **b** After the scanning, the 3D-reconstruction images are available on the screens. **c** A navigated tactile awl is used to establish the trajectories for the pedicle screws. **d** On the screens, both the navigated tactile awl (*arrow*) and the patient’s spine are shown in real time, and hence, the trajectories can be made under the guidance of the navigator

Firstly, a patient tracker is fixed to the spinal process (Fig. 1) followed by a 3D scanning using a C-arm (Fig. 2). After the scanning, the image data are transferred within 30 s from the 3D C-arm to the navigation workstation. As a result, the lumbar spine of the patient can be tracked by the navigation system in real time. Meanwhile, the navigated instruments are also being tracked.

**Fig. 3:**
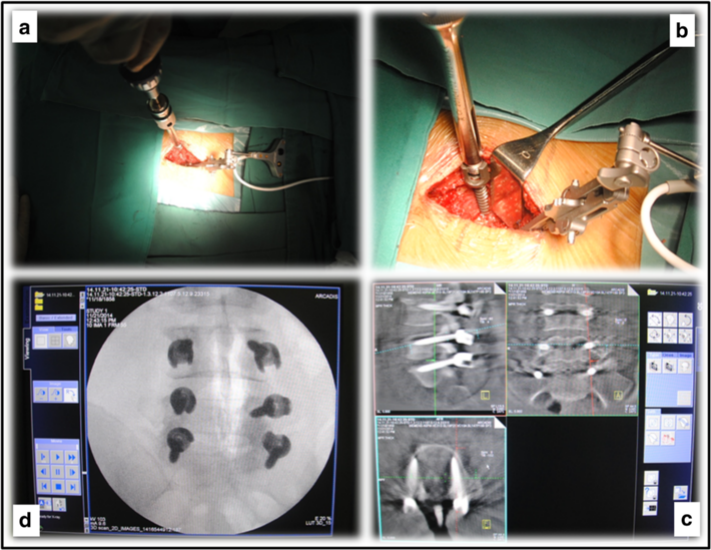
**a** When a trajectory has been established, a screw will be inserted. **b** The operator always has the visual of the screw during the course of screw insertion. **c**, **d**, Once all the six screws have been inserted, another round of 3D scanning will be performed to check the positions of the screws. After the scanning, the screws are shown in 3D-reconstruction images, and the positions of which can be clearly seen

Secondly, under the guidance of the navigator, six pedicle screws (multi-axial, 6.5-mm diameter) are inserted one by one. When a screw is being inserted, the muscles are pulled laterally and the operator always has the visual of the entry point (Fig. 3). Once all the six pedicle screws have been inserted, another 3D scanning is usually performed to check the position of each screw. If all the screws have shown to be well placed, the patient tracker is removed from the spinal process.

**Fig. 4:**
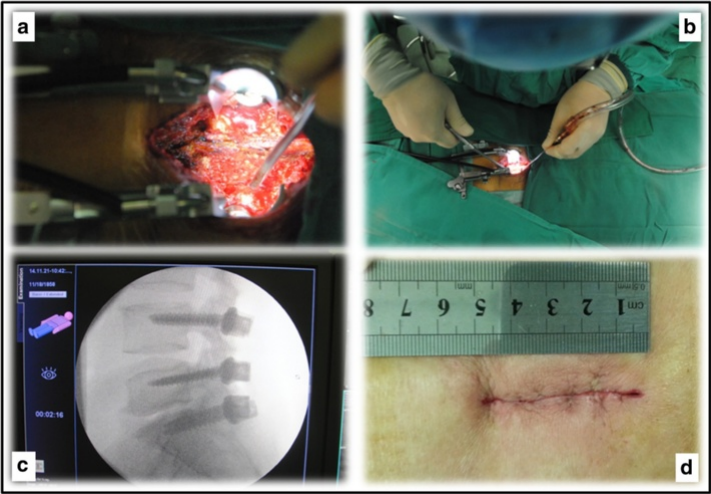
**a** A retractor is inserted to give the operator the visual of the laminas. **b** Decompression can be performed under direct vision. **c**, **d** The length of the incision is around 5 cm after closure

Lastly, a retractor is inserted to give the operator the visual of the laminas, by which decompression, discectomy, cage insertion, bone grafting, rod instrumentation, and screw nuts locking are performed (Fig. 4). Circumferential decompression of the dural sac and nerve roots was completed by removal of the lateral part of the lamina of the two vertebrae, and was considered satisfactory until only the middle pedicle remained visible. Posterolateral fusion using both autograft and allograft was performed in every case.

### Navigated PLF versus conventional PLF

A comparative study was performed between navigated PLF and conventional PLF. Forty patients who were indicated for 2-level lumbar fusion were included and randomized to either navigated PLF group or open-PLF group. Blood loss, operation time, incision length, complications, bed rest period, and length of hospitalization were recorded. Oswestry Disability Index (ODI) scoring was also performed for each patient before surgery, 3 months after surgery, and 2 years after surgery.

### Statistical analysis

The distributions of variables were presented as means ± standard deviation. *t* test and chi-square test were then used to detect the difference in each parameter between the two groups. Significance level was defined as 0.05. The statistical analyses were performed using STATA 11.0 software (Stata Corp., College Station, TX).

## Results

Forty patients were included and randomized to either navigated PLF or conventional-PLF group. All the patients were followed for at least 24 months. The demographic data were compared between the two groups (Table 1). The results showed that there was no significant difference between the two groups in terms of age, height, and weight.

**Table 1:** Comparison of demographic data between the two groups

	Navigated PLF group (*n* = 20)	Conventional PLF group (*n* = 20)	*p* value
Mean age (years)	64.7 ± 11.9	62.9 ± 9.6	0.592
Gender (M/F)	9/11	6/14	0.514
Height (cm)	160.6 ± 8.5	161.8 ± 7.6	0.641
Weight (kg)	63.4 ± 9.9	63.0 ± 10.3	0.889
Fusion level			
L3–L5 (no. of patients)	12	12	N/A
L4–S1 (no. of patients)	8	8	

*T* test or chi-square test was performed between the two groups; N/A - Not applicable

*p < 0.05

The operative data were compared between the two groups (Table 2). The incision length was significantly shorter in the navigated PLF group than in the conventional PLF group (4.8 vs. 10.9 cm, *p* = 0.001). Accordingly, the blood loss was also significantly less in the navigated PLF group than in the conventional PLF group (209.0 vs. 334.0 ml, *p* = 0.047). There was no significant difference in total operation time between the two groups (160.7 vs. 144.4 min, *p* = 0.116).

**Table 2:** Comparison of operative data between the two groups

	Navigated PLF group (*n* = 20)	Conventional PLF group (*n* = 20)	*p* value
Incision length (cm)	4.8 ± 0.4	10.9 ± 1.2	0.001*
Blood loss (ml)	209.0 ± 109.2	334.0 ± 248.7	0.047*
Operative time			
Total time (min)	160.7 ± 40.5	144.4 ± 20.8	0.116
Exposure (min)	35.2 ± 15.1	30.9 ± 11.5	0.319
Screw placement (min)	25.0 ± 6.4	26.7 ± 10.5	0.527
3D scanning (min)	11.8 ± 2.7	0 ± 0	N/A
Decompression (min)	76.6 ± 27.0	60.3 ± 15.4	0.024*
Closure (min)	12.2 ± 4.7	26.5 ± 5.6	0.001*

*T* test was performed between the two groups; N/A - Not applicable

*p < 0.05

**Fig. 5:**
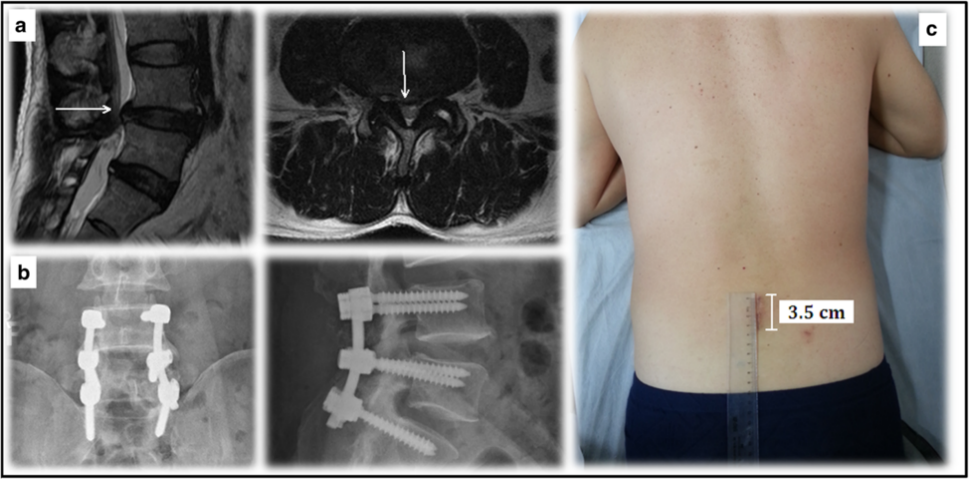
**a** MRI images showed disc herniation at L4/5 and L5/S1 levels (*arrows*). **b** A navigated PLF was performed. **c** The length of incision decreased from 4 to 3.5 cm in the 2 years after surgery

The postoperative data were compared between the two groups (Table 3). The navigated PLF group showed significantly shorter length of hospital stay, less postoperative blood loss, and less time to mobilization compared to the conventional PLF group. We also found that the incision length decreased with time in both groups (Fig. 5). The incision length decreased averagely from 4.8 to 4.3 cm in the navigated PLF group and from 10.9 to 10.3 cm in the conventional PLF group.

**Table 3:** Comparison of postoperative data between the two groups

	Navigated PLF group (*n* = 20)	Conventional PLF group (*n* = 20)	*p* value
Incision length at final follow-up (cm)	4.3 ± 0.3	10.3 ± 1.3	0.001*
Postoperative blood loss			
1st day (ml)	240.0 ± 91.8	359.5 ± 174.2	0.010*
2nd day (ml)	137.0 ± 63.8	177.5 ± 73.4	0.070
3rd day (ml)	60.5 ± 38.8	125.3 ± 130.3	0.040*
Total (ml)	437.5 ± 144.4	662.3 ± 320.2	0.007*
Time to mobilization (days)	2.2 ± 0.5	4.2 ± 0.4	0.001*
Length of hospital stay (days)	9.2 ± 1.8	12.5 ± 2.2	0.014*

*T* test was performed between the two groups

*p < 0.05

**Fig. 6:**
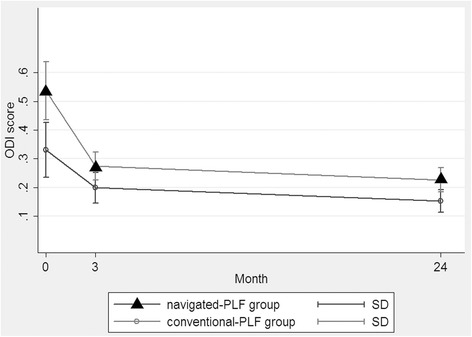
The ODI score improved significantly in the both groups immediately after surgery, and maintained well in the following 2 years

The clinical outcomes were compared between the two groups (Fig. 6). The ODI score improved significantly in the both groups immediately after surgery, and maintained well in the following 2 years.

The complications occurred in the two groups are listed in Table 4. One patient in the conventional PLF group underwent revision surgery for screw malposition on the seventh day after surgery. One patient in the navigated PLF group underwent revision surgery for hematoma on the tenth day after surgery.

**Table 4:** Complications occurred in the two groups

	Navigated PLF group (no.[%]) (*n* = 20)	Conventional PLF group (no.[%]) (*n* = 20)
Screw malposition	0	1 (5 %)
Cage migration	0	0
Dural tear	0	1 (5 %)
Hematoma	1 (5 %)	0
Superficial infection	1 (5 %)	1 (5 %)
Revision surgery	1 (5 %)	1 (5 %)

## Discussion

### Navigated PLF versus conventional PLF

As shown by the results, the navigated PLF showed several advantages compared to the conventional PLF. The navigated PLF group showed significantly less intraoperative blood loss. This finding is in accordance with the previous studies. As shown in Table 5, the average intraoperative blood loss of 2-level conventional PLIF were reported to be 612 ml (by Sakaura [10]) and 1277.6 ml (by Hioki [11]), while the average intraoperative blood loss of 2-level minimally invasive TLIF was much less, which was reported to be 206 ml (by Scarone [12]). This could be due to the navigated PLF’s having smaller incision and less soft-tissue destruction. Furthermore, the current study showed that the navigated PLF procedure had less postoperative blood loss in comparison with the conventional PLF (662.3l vs. 437.5 ml, *p* = 0.007). Regarding operation time, both of the previous and current studies showed similar results, the time consumption of minimally invasive and conventional lumbar fusion were comparable (Table 5).

**Table 5:** Operation time and blood loss for 2-level lumbar fusion

Author	Operation time (min)	Intraoperative blood loss (ml)
Sakaura [10] (PLIF)	218	612
Hioki [11] (PLIF)	301.8	1277.6
Scarone [12] (MIS-TLIF)	249.6	206
Current study (navigated PLF)	160.7	209.0
Current study (conventional PLF)	144.4	334.0

*MIS* minimally invasive surgery

As for clinical outcomes, the both groups showed significant ODI-score improvements, which also compared well with the previous studies [3–9].

In the current study, the average length of incision of the conventional PLF was two times of that of the navigated PLF (10.9 vs. 4.8 cm, *p* = 0.001), which was one of the major superiorities of the navigated PLF. In addition, we found that the incision length decreased with time. At the final follow-up, the incision length had decreased averagely from 4.8 to 4.3 cm in the navigated PLF group and from 10.9 to 10.3 cm in the conventional PLF group.

### Navigated PLF versus other minimally invasive techniques

Several minimally invasive procedures have been developed in order to lessen the approach related morbidity. Schwender et al. [1] presented the first clinical series reporting on minimally invasive transforaminal lumbar interbody fusion (MiTLIF). A paramedian, muscle-sparing approach was performed through a tubular retractor. Facetectomy, discectomy, and interbody cage insertion through the tube were performed. Bilateral percutaneous pedicle screw-rod placement was then accomplished with the Sextant system. Scheufler et al. [3] reported their clinical study on percutaneous transforaminal lumbar interbody fusion (pTLIF). Decompression, discectomy, and interbody cage insertion were performed through tubular retractors followed by percutaneous pedicle screw-rod fixation. Isaacs et al. [2] developed microendoscopic transforaminal lumbar interbody fusion (METLIF). Hemilaminectomy, unilateral facetectomy, and microdiscectomy were performed using microendoscopy-assisted lumbar fusion through a working channel. Bilateral percutaneous pedicle screws were then inserted.

All the minimally invasive techniques mentioned above require totally four to seven incisions, and one of these incisions has to be around 4 cm (ranging from 3.5 to 4.5 cm) so that an expandable retractor can be accommodated. The present technique requires only a single 5-cm incision, and hence greatly decreases the number of incisions, which is one of the superiorities of navigated PLF over the other minimally invasive techniques. However, small skin incision does not necessarily mean small muscle injury. Navigated PLF in the current study still involves muscle detachment and ligamentous disruption, which should be improved in the future. Small skin incision could be a problem for navigation, because the patient tracker could be moving when the wound is being retracted laterally. As such, patient tracker must be fixed firmly and care must be taken when retracting the wound.

The length of incision could be further decreased if the pedicle-screw direction was well designed [13].

Another important advantage of navigated PLF is that the pedicle screws are inserted under the guidance of the infra-red navigators, which not only make the procedure safer but also completely avoid the operation personnel’s exposure to radiation.

## Conclusions

Compared to conventional PLF, navigated PLF proved to be superior with regard to incision length, blood loss, time to mobilization, and shorter length of hospital stay.

## References

[1] Schwender JD, Holly LT, Rouben DP (2005). Minimally invasive transforaminal lumbar interbody fusion (TLIF): technical feasibility and initial results. J Spinal Disord Tech.

[2] Isaacs RE, Podichetty VK, Santiago P (2005). Minimally invasive microendoscopy-assisted transforaminal lumbar interbody fusion with instrumentation. J Neurosurg Spine.

[3] Scheufler KM, Dohmen H, Vougioukas VI (2007). Percutaneous transforaminal lumbar interbody fusion for the treatment of degenerative lumbar instability. Neurosurgery.

[4] Park Y, Ha JW (2007). Comparison of one-level posterior lumbar interbody fusion performed with a minimally invasive approach OR traditional open approach. spine.

[5] Dhall SS, Wang MY, Mummaneni PV (2008). Clinical and radiographic comparison of mini-open transforaminal lumbar interbody fusion with open transforaminal lumbar interbody fusion in 42 patients with long-term follow-up. J Neurosurg Spine.

[6] Fan S, Hu Z, Zhao X (2009). Multifidus muscle changes and clinical effects of one-level posterior lumbar interbody fusion: minimally invasive procedure versus conventional open approach. Eur Spine J.

[7] Schizas C, Tzinieris N, Tsiridis E (2009). Minimally invasive versus open transforaminal lumbar interbody fusion: evaluating initial experience. Int Orthop.

[8] Kotani Y, Abumi K, Ito M, Sudo H (2012). Mid-term clinical results of minimally invasive decompression and posterolateral fusion with percutaneous pedicle screws versus conventional approach for degenerative spondylolisthesis with spinal stenosis. Eur Spine J.

[9] Wang J, Zhou Y, Zhang ZF (2010). Comparison of one-level minimally invasive and open transforaminal lumbar interbody fusion in degenerative and isthmic spondylolisthesis grades 1 and 2. Eur Spine J.

[10] Sakaura H, Yamashita T, Miwa T (2013). Outcomes of 2-level posterior lumbar interbody fusion for 2-level degenerative lumbar spondylolisthesis. J Neurosurg Spine.

[11] Hioki A, Miyamoto K, Kodama H (2005). Two-level posterior lumbar interbody fusion for degenerative disc disease: improved clinical outcome with restoration of lumbar lordosis. Spine J.

[12] Scarone P, Lepeintre JF, Bennis S (2009). Two-levels mini-open transforaminal lumbar interbody fusion: technical note. Minim Invasive Neurosurg.

[13] Reinshagen C, Ruess D, Walcott BP (2015). A novel minimally invasive technique for lumbar decompression, realignment, and navigated interbody fusion. J Clin Neurosci.

